# Overview of 15-year severe combined immunodeficiency in the Netherlands: towards newborn blood spot screening

**DOI:** 10.1007/s00431-015-2518-4

**Published:** 2015-04-01

**Authors:** Anne P. J. de Pagter, Robbert G. M. Bredius, Taco W. Kuijpers, Jelco Tramper, Mirjam van der Burg, Joris van Montfrans, Gertjan J. Driessen, on behalf of the Dutch Working Party for Immunodeficiencies

**Affiliations:** 1 Department of Pediatrics, Erasmus MC, Sophia Children’s Hospital, Rotterdam, The Netherlands; 2 Department of Pediatrics, Leiden University Medical Center, Leiden, The Netherlands; 3 Department of Pediatrics, Academic Medical Center, Amsterdam, The Netherlands; 4 Department of Immunology, Erasmus Medical Center, Rotterdam, The Netherlands; 5 Department of Pediatrics, University Medical Center Utrecht, Wilhelmina Children’s Hospital, Utrecht, The Netherlands; 6 Department of Pediatric, Division Infectious Disease and Immunology, Erasmus MC, Sophia Children’s Hospital, P.O. Box 2060, 3015 GJ Rotterdam, The Netherlands

**Keywords:** Severe combined immunodeficiency, Primary immunodeficiency, Infection, Viral, Bacterial, Stem cell transplantation

## Abstract

Severe combined immune deficiency (SCID) is a fatal primary immunodeficiency usually presenting in the first months of life with (opportunistic) infections, diarrhea, and failure to thrive. Hematopoietic stem cell transplantation (HSCT) and gene therapy (GT) are curative treatment options. The objective of the study was to assess the morbidity, mortality, and diagnostic and therapeutic delay in children with SCID in the Netherlands in the last 15 years. These data may help to judge whether SCID should be considered to be included in our national neonatal screening program. In the period 1998–2013, 43 SCID patients were diagnosed in the Netherlands, 11 of whom were atypical SCID (presentation beyond the first year). The median interval between the first symptom and diagnosis was 2 months (range 0–1173 months). The total mortality was 42 %. In total, 32 patients were treated with HSCT of whom 8 were deceased. Nine patients died due to severe infectious complications before curative treatment could be initiated.

*Conclusion*: Because of a high mortality of patients with SCID before HSCT could be initiated, only a national newborn screening program and pre-emptive HSCT or GT will be able to improve survival of these patients.

**Table:** 

“**What is known**” • *SCID is a fatal disease if a curative hematopoietic stem cell transplantation cannot be performed in time*. • *Newborn screening for SCID enables early diagnosis in the asymptomatic phase*.
“**What is new**” • *Nine out of 43 SCID patients in the Netherlands* *died due to severe infectious complications before curative treatment could be initiated*. • *Only newborn screening and pre*-*emptive curative therapy will improve survival of children with SCID in the Netherlands*.

“**What is known**”

• *SCID is a fatal disease if a curative hematopoietic stem cell transplantation cannot be performed in time*.

• *Newborn screening for SCID enables early diagnosis in the asymptomatic phase*.

“**What is new**”

• *Nine out of 43 SCID patients in the Netherlands* *died due to severe infectious complications before curative treatment could be initiated*.

• *Only newborn screening and pre*-*emptive curative therapy will improve survival of children with SCID in the Netherlands*.

## Introduction

Severe combined immune deficiency (SCID) is a life-threatening primary immunodeficiency usually presenting in the first months of life with (opportunistic) infections, protracted diarrhea, and failure to thrive [11]. Patients with SCID are highly susceptible to severe and recurrent infections and usually do not survive infancy unless provided with immune reconstituting treatments, such as hematopoietic stem cell transplantation (HSCT), PEG-ADA, or gene therapy (GT) [11]. Although infections are common in the pediatric population, an immunodeficiency such as SCID should be suspected if infections are recurrent, severe, or caused by an opportunistic pathogen.

The immunodeficiency in SCID patients is characterized by severe defects in cellular and humoral immunity. The hallmark is the absence of T cells, with a primary or secondary dysfunction of B cells, leading to hypogammaglobulinemia.

Different pathophysiological mechanisms cause absence or dysfunction of T cells, depending on the genetic defect. If the genetic defect affects the development of T cells as well as B cells, the disease is classified as T–B− SCID. If only T cells are affected, with a secondary dysfunction of B cells, patients are characterized as T–B+ SCID. Multiple genetic defects in more than 30 different genes can cause SCID, and another 30 genes can cause other forms of T cell lymphopenia.[1]

Reduced thymopoiesis can be diagnosed in newborn dried blood spots by high-throughput analysis of T cell receptor excision circles (TRECs), enabling neonatal screening for SCID.[4, 8, 10]. Newborn screening for SCID has been introduced with success in parts of the USA [7]. The Clinical and Laboratory Standards Institute (CLSI) recently published an international guideline to use newborn bloodspots for SCID screening by measurement of TRECs [6]. Subsequent lymphocyte immunophenotyping and genetic analysis can confirm the diagnosis of SCID in case low TRECs and T cell lymphopenia are detected.

The aim of this study was to determine if SCID is a suitable candidate for implementation in the newborn screening program in the Netherlands [12]. Therefore, we investigated the presenting symptoms, morbidity, delay in diagnosis and treatment, and the mortality of all SCID patients over the last 15 years in the Netherlands.

## Material and methods

In this retrospective cohort study, all SCID patients who were born in the Netherlands between January 1998 and January 2013 (*n* = 43) were included. SCID patients who underwent HSCT were selected from the European Group for Blood and Marrow Transplantation (EBMT) and from the National Registry as part of the European Society for immunodeficiencies (ESID) databases. The other patients were identified by reviewing medical records and local patient lists in each tertiary referral center for clinical pediatric immunology. Detailed information about HSCT treatment regimens was collected in the LUMC and UMC Utrecht (HSCT centers in the Netherlands).

Data collection included general characteristics, family history, consanguinity, diagnosis, morbidity (type of infections, frequency and severity of infections, and other symptoms like autoimmunity and failure to thrive), laboratory data, (antimicrobial) treatments, and mortality. In addition, we explored the delay between date of birth, date of first symptoms (FS), date of diagnosis, and date of therapy or death. Atypical SCID was defined as diagnosed >1 year after birth. Typical SCID was defined as diagnosed <1 year after birth. Data were collected in an anonymized way. Ethical approval was given by institutional review board.

Differences in patient groups were analyzed using chi-square analyses. All analyses were performed using software program SPSS 21.0 (SPSS Inc., Chicago, IL, USA).

## Results

**Fig. 1:**
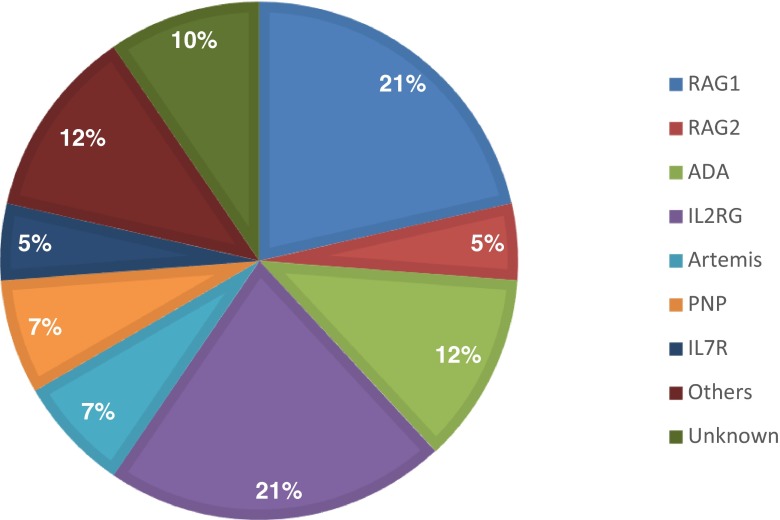
Distribution of SCID patients (*n* = 43) based on genetic diagnosis

From 1998–2013, 43 patients with SCID (23 boys and 20 girls) were identified in the Netherlands. On a total population of ~180,000 newborns/year, the incidence in this period was approximately 1:63,000. Eleven patients were diagnosed with an atypical form of SCID. In 11 patients, consanguinity was present. Patient characteristics, immunophenotyping, and genetics are described in Table 1 and Fig. 1.

**Table 1:** Patient characteristics

Patient no.	Symptoms	Gene defect	CD3+	CD4+	CD8+	NK+	CD19+	Age at diagnosis (months)	Alive	HSCT
1	Gastr	RAG1	−	−	−	+	−	67	−	−
2	Resp, Gastr	RAG1	−	−	−	+	+	37	+	+
3	Syst, Resp, Gastr	RAG1	−	−	−	+	−	6	+	+
4	Resp, Gastr	RAG1	−	−	−	+	−	3	−	+
5	Other	RAG1	−	−	−	+	−	3	+	+
6	Syst, Gastr, Other	RAG1	+	+	+	−	−	2	−	−
7	Resp, Other	RAG1	−	−	−	−	−	11	−	−
8	Resp, Gastr	RAG1	-	−	−	+	−	4	−	+
9	Syst, Gastr	RAG1	−	+	−	−	−	9	−	−
10	Syst, Resp, Gastr	RAG2	−	−	−	+	−	4	+	+
11	Other	RAG2	−	−	−	+	−	5	+	+
12	Resp	ADA	−	−	−	+	−	31	−	−
13	Other	ADA	−	−	−	−	−	1	+	^a^
14	Gastr	ADA	−	−	−	−	−	1	+	+
15	Other	ADA	+	−	+	+	+	1	+	+
16	Resp, Gastr	ADA	−	−	−	−	−	1	−	−
17	Resp, Gastr	IL2RG	−	−	−	−	−	7	+	+
18	Resp, Gastr Other	IL2RG	+	−	+	+	+	64	−	+
19	Resp, Gastr	IL2RG	−	−	−	+	+	5	+	+
20	Fam	IL2RG	−	−	+	+	+	0	+	+
21	Resp	IL2RG	+	−	−	−	+	4	+	+
22	Resp	IL2RG	+	−	+	−	+	13	+	+
23	Resp	IL2RG	−	−	−	+	+	17	+	+
24	Other	IL2RG	−	−	−	−	+	3	+	+
25	Fam	IL2RG	−	−	−	−	+	0	−	^a^
26	Resp	Artemis	−	−	+	−	+	157	−	+
27	Syst, Resp	Artemis	−	−	−	−	−	75	+	+
28	Syst, Other	Artemis	−	−	−	+	−	3	−	+
29	Resp	PNP	−	−	−	−	+	19	+	+
30	Syst, Resp, Gastr	PNP	−	−	−	−	−	32	+	+
31	Other	PNP	−	−	−	−	+	55	+	+
32	Other	RMRP	+	−	+	+	+	9	−	+
33	Other	IL7R	−	−	−	−	+	6	+	+
34	Syst, Gastr	IL7R	−	−	−	+	+	3	+	+
35	Fam	AK-2	−	−	−	−	−	0	+	+
36	Syst, Gastr	T7q-20q	−	−	−	−	+	4	+	+
37	Resp	CD3E	−	−	−	+	+	1	−	−
38	Syst, Resp, Gastr	LIG4	−	−	−	−	−	8	−	−
39	Syst, Resp, Gastr	ZAP70	−	−	−	+	+	80	+	+
40	Resp, Gastr	Unknown	−	−	−	−	−	7	−	+
41	Resp	Unknown	−	−	−	−	+	3	−	+
42	Gastr	Unknown	−	−	−	+	+	2	−	−
43	Other	Unknown	−	−	−	+	+	5	+	+

*Syst* systemic infections (e.g., sepsis), *Resp* respiratory infections, *Gastr* gastrointestinal symptoms/failure to thrive, *Other* skin, other, *Fam* positive family history of SCID.

^a^Gene therapy

**Fig. 2:**
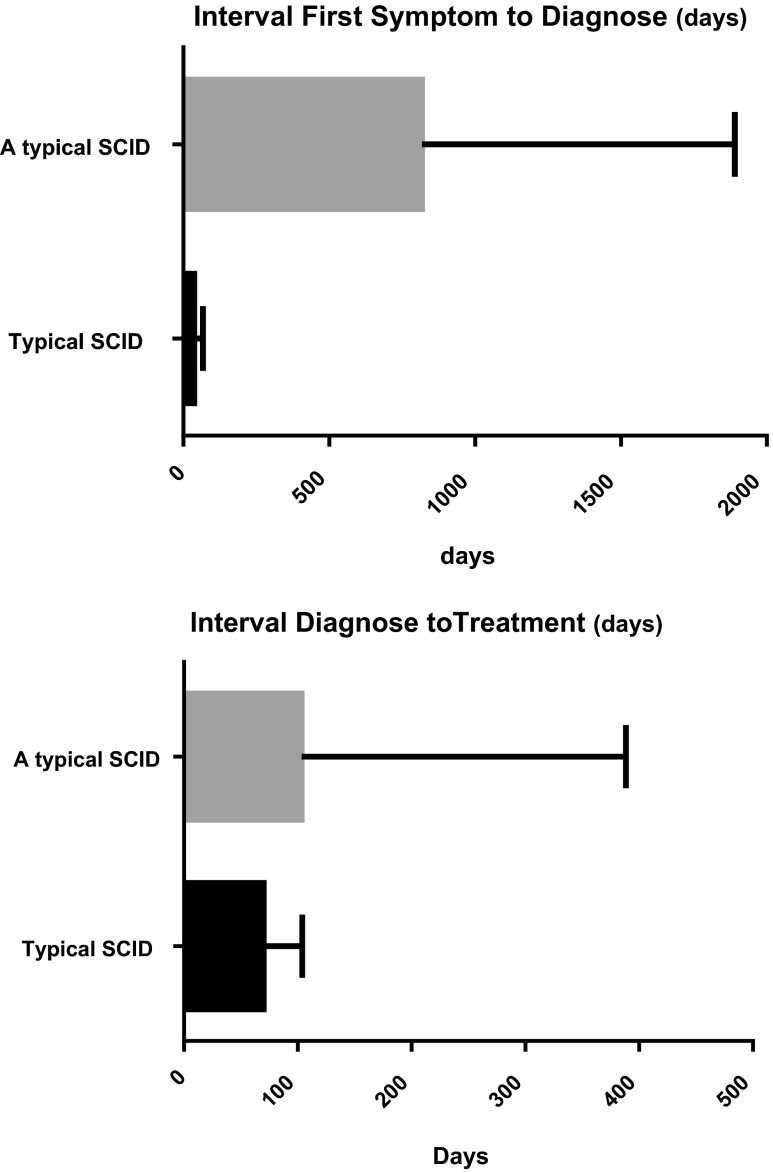
Delay between first symptoms to diagnosis, and diagnosis to treatment for atypical and typical SCID patients. Boxplots show median (*box*) and interquartile range (*line*)

Median follow-up of patients was 66 months (range 1–198 months). Figure 2 shows the intervals between first symptoms and diagnosis, and diagnosis and treatment. Delay of diagnosis (median 27 months after first symptoms) was more pronounced in atypical SCID compared to typical SCID (median 2 months after first symptoms) (Fig. 2).

Prior to curative treatment, 22 patients suffered from systemic viral/bacterial infections and 25 suffered from opportunistic infections. Eleven of 25 patients with opportunistic infections had *Pneumocystis jiroveci* pneumonia, 8 had systemic cytomegalovirus infection, and 6 had BCG-itis. Of these patients, 8 were deceased at a median of 12 days (range 0–88 days) after initial presentation with a fulminant infections despite antimicrobial treatment (Table 2). There was no association between delay in diagnosis and mortality (*p* = 0.269) in the typical SCID patients. In the atypical SCID group, we found symptoms of auto-immunity (hemolytic anemia) in 5 of 11 patients.

**Table 2:** Infections in SCID patients prior to treatment

	HSCT or GT (*n* = 34)^a^	No HSCT or GT (*n* = 9)^b^	*p* value
Bacterial sepsis	3	8	0.001
Pneumonia			
*P. jiroveci*	9	2	
Other	13	6	0.237
Systemic viral infection			
Adenovirus	3	1	
Cytomegalovirus	5	3	
Other	5	0	1.01
BCG-itis/TB	5	1	0.998
*Cryptosporidium*	2	2	0.189
*Pseudomonas*	2	1	0.510

*HSCT* hematopoietic stem cell transplantation, *GT* gene therapy

^a^Seven patients suffered from >1 infection

^b^Five patients suffered from >1 infection

Three patients were diagnosed prior to infectious complications in the first week of life because of a positive family history. In one patient, prematurity with lymphopenia and congenital deafness was present and cartilage-hair-hypoplasia with SCID was diagnosed. Two of these three patients underwent HSCT and one patient received gene therapy. This patient was deceased due to secondary malignancy following initially successful gene transfer [15].

**Fig. 3:**
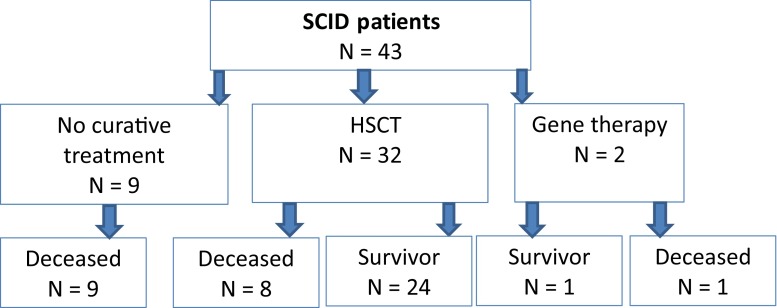
Outcome of 43 diagnosed SCID patients. *HSCT* hematopoietic stem cell transplantation

In total, 34 patients received curative treatment, of whom 2 received gene therapy (Fig. 3). Eleven of 24 survivors showed infectious complications after HSCT and 2 of 24 patients had allo-reactive complications. Twentyfour of the 32 patients were successfully transplanted. One ADA SCID patient was successfully treated with gene therapy.

## Discussion

This retrospective analysis of SCID patients in the Netherlands shows that morbidity and mortality were high. The incidence may be an underestimation due to non-diagnosed infants, deceased due to severe infection. The majority of deaths were associated with severe infections. Given the fact that early identification newborn screening is feasible, these data suggest that a significant number of SCID cases can be cured by early HSCT or gene therapy, preceded by appropriate anti-infectious prophylaxis after implementation of newborn screening for SCID.

The occurrence of the first symptoms of SCID at 2–3 months after birth was comparable with observations of others [5, 11]. We observed a relatively high proportion of patients with atypical SCID in our cohort [13]. These patients had severe infections and high mortality rates. A clinical presentation with milder or non-opportunistic infections and/or auto-immunity explained the longer delay in diagnosis.

Importantly, our data indicate that even if SCID is diagnosed early after the initial presenting symptoms, mortality cannot be prevented in a considerable proportion of SCID patients because of a fulminant course of the presenting infection. Many of these patients were diagnosed with bacterial sepsis. So, the only way to improve the outcome is to diagnose these patients by a newborn screening program for SCID before symptoms occur. The asymptomatic patients who received HSCT early in life were successfully transplanted, as described by others [3]. However, in patients surviving until HSCT, mortality due to pre-HSCT infections was still considerable, in line with other studies [2, 3]. Newborn diagnosis and early treatment of SCID in the asymptomatic phase improves the outcome by reduction of infections and improved overall survival after HSCT [2, 3]. Recently, Pai et al. reported excellent survival rates of 94 % in SCID patients transplanted in the first 3.5 months (*n* = 68) compared to 66 % in patients who received HSCT after 3.5 months of age (*n* = 172)[9].

Considering the prerequisites for neonatal screening [12], we showed that in most patients, an early asymptomatic phase is present, enabling identification in this pre-symptomatic episode and in good clinical condition. These data support the idea that newborn screening for SCID will also be useful to prevent death and severe complications in the Dutch situation and comparable countries [7].

In conclusion, this study shows a high morbidity and mortality in SCID patients in the Netherlands, irrespective of age at diagnosis, diagnostic delay and intensive treatment of infections and supportive care preceding HSCT. We further found a high proportion of patients with atypical presentation of SCID, which warrants a high level of suspicion in patients with infections and immune dysregulation. Because most patients had an asymptomatic phase and because early HSCT has a good prognosis in such patients, our findings strongly support the implementation of newborn screening for SCID in the Netherlands.

## Abbreviations

EBMT: European Group for Blood and Marrow Transplantation
ESID: European Society for Immunodeficiencies
GT: Gene therapy
HSCT: Hematopoietic stem cell transplantation
SCID: Severe combined immunodeficiency

## References

[1] Al-Herz W, Bousfiha A, Casanova JL, Chapel H, Conley ME, Cunningham-Rundles C, Etzioni A, Fischer A, Franco JL, Geha RS, Hammarstrom L, Nonoyama S, Notarangelo LD, Ochs HD, Puck JM, Roifman CM, Seger R, Tang ML (2011). Primary immunodeficiency diseases: an update on the classification from the international union of immunological societies expert committee for primary immunodeficiency. Front Immunol.

[2] Antoine C, Muller S, Cant A, Cavazzana-Calvo M, Veys P, Vossen J, Fasth A, Heilmann C, Wulffraat N, Seger R, Blanche S, Friedrich W, Abinun M, Davies G, Bredius R, Schulz A, Landais P, Fischer A (2003). Long-term survival and transplantation of haemopoietic stem cells for immunodeficiencies: report of the European experience 1968-99. Lancet.

[3] Brown L, Xu-Bayford J, Allwood Z, Slatter M, Cant A, Davies EG, Veys P, Gennery AR, Gaspar HB (2011). Neonatal diagnosis of severe combined immunodeficiency leads to significantly improved survival outcome: the case for newborn screening. Blood.

[4] Buckley RH (2012). The long quest for neonatal screening for severe combined immunodeficiency. J Allergy Clin Immunol.

[5] Chan A, Scalchunes C, Boyle M, Puck JM (2011). Early vs. delayed diagnosis of severe combined immunodeficiency: a family perspective survey. Clin Immunol.

[6] Institute CaLS. Newborn blood spot screening for severe combined immunodeficiency by measurement of T-cell receptor excision circles; Approved guideline. *CLSI NBS06-A* 2013

[7] Kwan A, Church JA, Cowan MJ, Agarwal R, Kapoor N, Kohn DB, Lewis DB, McGhee SA, Moore TB, Stiehm ER, Porteus M, Aznar CP, Currier R, Lorey F, Puck JM (2013) Newborn screening for severe combined immunodeficiency and T-cell lymphopenia in California: results of the first 2 years. J Allergy Clin Immunol 132:140–150 10.1016/j.jaci.2013.04.024 PMC3759317 23810098

[8] McGhee SA, Stiehm ER, Cowan M, Krogstad P, McCabe ER (2005). Two-tiered universal newborn screening strategy for severe combined immunodeficiency. Mol Genet Metab.

[9] Pai SY, Logan BR, Griffith LM, Buckley RH, Parrott RE, Dvorak CC, Kapoor N, Hanson IC, Filipovich AH, Jyonouchi S, Sullivan KE, Small TN, Burroughs L, Skoda-Smith S, Haight AE, Grizzle A, Pulsipher MA, Chan KW, Fuleihan RL, Haddad E, Loechelt B, Aquino VM, Gillio A, Davis J, Knutsen A, Smith AR, Moore TB, Schroeder ML, Goldman FD, Connelly JA, Porteus MH, Xiang Q, Shearer WT, Fleisher TA, Kohn DB, Puck JM, Notarangelo LD, Cowan MJ, O'Reilly RJ (2014). Transplantation outcomes for severe combined immunodeficienty, 2000-2009. N Engl J Med.

[10] Puck JM, SCID Newborn Screening Working Group (2007). Population-based newborn screening for severe combined immunodeficiency: steps toward implementation. J Allergy Clin Immunol.

[11] van der Burg M, Gennery AR (2011). Educational paper: the expanding clinical and immunological spectrum of severe combined immunodeficiency. Eur J Pediatr.

[12] Wilson JM, Jungner YG (1968). Principles and practice of mass screening for disease. Bol Oficina Sanit Panam.

[13] Yee AS, De Ravin S, Elliot E, Ziegler JB (2008). Severe combined immunodeficiency: a national surveillance study. Pediatr Allergy Immunol.

